# Paediatric palliative home care in areas of Germany with low population density and long distances: a questionnaire survey with general paediatricians

**DOI:** 10.1186/1756-0500-5-498

**Published:** 2012-09-11

**Authors:** Kerstin Kremeike, Nina Eulitz, Saskia Jünger, Annette Sander, Max Geraedts, Dirk Reinhardt

**Affiliations:** 1 Netzwerk für die Versorgung schwerkranker Kinder und Jugendlicher e.V, Carl-Neuberg-Straße 1, 30625 Hannover, 30625, Germany; 2 Department of Paediatric Haematology and Oncology, Hannover Medical School, Carl-Neuberg-Straße 1, 30625 Hannover, 30625, Germany; 3 Department of Palliative Medicine, University Hospital of Bonn, Sigmund-Freud-Str. 25, 53127 Bonn, 53127, Germany; 4 Institute for Health Systems Research, Witten/Herdecke University, Alfred-Herrhausen-Straße 50, 58448 Witten, 58448, Germany

**Keywords:** Children, Palliative care, Paediatrician’s survey, Home care, Network, Prevalence

## Abstract

**Background:**

In 2007, the patient’s right to specialised palliative home care became law in Germany. However, childhood palliative care in territorial states with low patient numbers and long distances requires adapted models to ensure an area-wide maintenance. Actually, general paediatricians are the basic care providers for children and adolescents. They also provide home care. The aim of this study was to improve the knowledge about general paediatrician’s involvement in and contribution to palliative care in children.

**Findings:**

To evaluate the current status of palliative home care provided by general paediatricians and their cooperation with other paediatric palliative care providers, a questionnaire survey was disseminated to general paediatricians in Lower Saxony, a German federal state with nearly eight million inhabitants and a predominantly rural infrastructure. Data analysis was descriptive.

One hundred forty one of 157 included general paediatricians completed the questionnaire (response rate: 89.8%). A total of 792 children and adolescents suffering from life-limiting conditions were cared for by these general paediatricians in 2008. Severe cerebral palsy was the most prevalent diagnosis. Eighty-nine per cent of the general paediatricians stated that they had professional experience with paediatric palliative care.

Collaboration of general paediatricians and other palliative care providers was stated as not well developed. The support by a specialised team including 24-hour on-call duty and the intensification of educational programs were emphasised.

**Conclusions:**

The current regional infrastructure of palliative home care in Lower Saxony can benefit from the establishment of a coordinated network of palliative home care providers.

## Background

Paediatric palliative care aims to improve the quality of life for children and adolescents suffering from life-limiting conditions and their families [1]. Essential requirements for effective paediatric palliative care include a broad multi-disciplinary approach including available community resources. Families often prefer to care for their ill child at home [2]. Therefore, the existence of local home care providers is crucial [3].

Generalists play an important role in palliative care – in Germany as well as in other countries [4,5]. In the German health care system, general paediatricians provide medical home care for children and adolescents. Since they can make life-changing treatment decisions, optimal professional expertise, including the various aspects of palliative home care, is essential to providing appropriate support for patients and families [6,7].

General paediatricians working in private practice are supposed to be familiar with the basic principles of palliative care [8,9]; however, this may be hampered by marginal experience with palliative patients [10,11]. Heterogeneous life-limiting diseases and unpredictable clinical courses [12-14] are major causes of insecurity among general paediatricians when it comes to managing a palliative situation [11,15,16].

Lower Saxony is a federal state in north-western Germany with nearly eight million inhabitants, a predominantly rural infrastructure, and about 470 paediatricians working in private practice. Data on the prevalence of life-limiting conditions in the region are missing.

To date, paediatric palliative home care in Germany is characterised by a shortage of financial resources and insufficient staff [17]. In Lower-Saxony, a regional tailored concept of a comprehensive specialist paediatric palliative home care has been implemented, as anchored in the German social legislation in April 2007.

The aim of the study was to evaluate involvement in and contribution of general paediatricians in paediatric palliative care and their cooperation with other paediatric palliative care providers. The latter can be seen as crucial for adequate paediatric palliative care – especially in rural areas with low patient numbers and long distances like Lower Saxony, where huge distances complicate effective paediatric palliative care [18].

## Findings

### Methods

The survey was conducted between May and December 2009. Data on patients’ characteristics, management, and networking were asked going back to 2008. A standardised questionnaire was designed based on

an established instrument used in North-Rhine Westphalia (NRW), another German federal state [7,10];

the classification of life-limiting conditions first described by the Association for Children with Life Threatening or Terminal Conditions and their Families (ACT) (see Table 1) [19,20]; and

a review of the literature [11,15,21,22].

**Table 1:** **Classification of life-limiting conditions according to the Association for Children with Life Threatening or Terminal Conditions and their Families (ACT) **[19,20]

	
Group 1	Conditions for which treatment with curative intention is feasible but might fail, e.g., cancer or irreversible organ failure.
Group 2	Conditions for which there are long periods of intensive treatment aimed at prolonging good-quality life but for which premature death is anticipated, e.g., cystic fibrosis, muscular dystrophy.
Group 3	Progressive condition where treatment is exclusively palliative from the time of diagnosis and may extend over many years, e.g., metabolic disorders.
Group 4	Conditions with severe disability, often neurological, which although not progressive cause extreme vulnerability to health complications and where premature death is anticipated, e.g., severe cerebral palsy, chromosomal disorders.

The questionnaire developed by Juenger et al. in NRW was based on a qualitative exploration [10]. This instrument was adjusted for the purpose of our study by replacing a section on the role of the general paediatricians with questions on interdisciplinary professional networking and the transition from inpatient to outpatient care known as a particular challenge [23]. The modification was done to obtain more detailed information on the cooperation between general paediatricians and other paediatric palliative care providers, being a topic of special interest in areas with low patient numbers and long distances like Lower Saxony.

Furthermore, the original instrument was upgraded by introducing the ACT classification [19,20] at the beginning of the questionnaire, assuming that paediatricians could have difficulties considering all life-limiting conditions as such. Beyond that, year dates were added to all questions concerning patients’ characteristics, management, and networking to generate more detailed data.

The therewith-obtained questionnaire consisted of 25 questions addressing the following aspects:

Previous experience with and exposure to situations requiring paediatric palliative care

Interdisciplinary professional networking and the transition from inpatient to outpatient care

Potential difficulties and incentives specifically associated with the involvement of general paediatricians in paediatric palliative care [7,16,21]

Demographical data

The questionnaire used a homogeneous item-and-response format (six-point Likert scales) to evaluate potential difficulties and incentives; free-text options were given to specify the prior experience in paediatric palliative care, the cooperation with other care providers, possible barriers and incentives, and – in a separately formulated closing question – to give the opportunity for suggestions and supplementary notes.

The generated questionnaire (see Additional file 1 for the final version) was pilot-tested with nine general paediatricians working in private practice. Resulting from the pilot test, a more detailed oral introduction of the ACT categories was given during the following survey. Ethical approval for the study was given by the Ethics Committee of the Medical School Hannover.

In Germany, all paediatricians working in private practice are required to participate in a regional quality circle in order to meet the demands of continuing medical education. Therefore, the investigators (KK and NE) visited all 11 regional paediatric quality circles in Lower Saxony, explained the research goal, distributed the questionnaires, collected them after completion and were available for questions or comments. Participants took an average of 20 minutes to complete the questionnaire.

Descriptive analyses were performed using the Statistic Package for Social Sciences (SPSS) 18.0.

### Results

#### Sample

Out of 156 questionnaires distributed, 141 were completed. Sixteen paediatricians refused to answer the questionnaire for various reasons, resulting in a response rate of 89.8%.

More than one-third (53, 37.6%) of the participants worked in a mainly rural environment, and 56.7% (80) worked in an urban area. A minority (8, 5.7%) did not specify the region they were working in.

The average age of the participants was 51 years (range 33 – 68 years), with 86 (61.0%) of them being male. The paediatricians reported a median work experience of 11 years (range 1 to 31). The mean number of patient contacts was 1400 per quarter-year. More than half of the paediatricians (82, 58.2%) worked in their practice on their own, and 58 (41.1%) worked in a group practice. The characteristics of the participating paediatricians and the reported patients are comparable to the total groups in Lower Saxony [24].

#### Previous experience with and exposure to situations requiring paediatric palliative care

Most of the participants (128/141, 90.8%) stated that they have had experience with palliative care, and 87 (61.7%) had taken care of children in the terminal phase of a life-limiting disease at home.

In 2008, the paediatricians were involved in the management of 792 children suffering from life-limiting conditions; 38 (4.8%) of these children died in the same year. The majority of children suffered from a condition that could be classified as ACT group 4 [19,20] (Table 1); most of them were afflicted by severe cerebral palsy. Table 2 shows the distribution of the stated conditions according to ACT groups and the most frequent diagnosis. Compared to the results in NRW, the distribution of conditions between the ACT groups shows significant differences for the ACT groups 1, 3, and 4.^a^

**Table 2:** Conditions stated by the paediatricians according to ACT groups and main diagnosis (2008)

	**Number of patients (percentages of total)**	**Main diagnosis**	**Number of patients (percentages within the ACT Group)**
ACT group 1	151 (19.1%)	Malignant diseases, Vitium Cordis	80 (53.0%), 51 (33.8%)
ACT group 2	146 (18.4%)	Cystic fibrosis, Duchenne muscular dystrophy	91 (62.3%), 51 (33.8%)
ACT group 3	91 (11.5%)	Metabolic disorders	66 (72.5%)
ACT group 4	358 (45.2%)	Cerebral palsy, Chromosomal disorders	209 (57.7%), 73 (20.4%)
Classification not possible	46 (5.8%)	Diverse syndromes	20 (30.3%)
Total	792		

**Figure 1:**
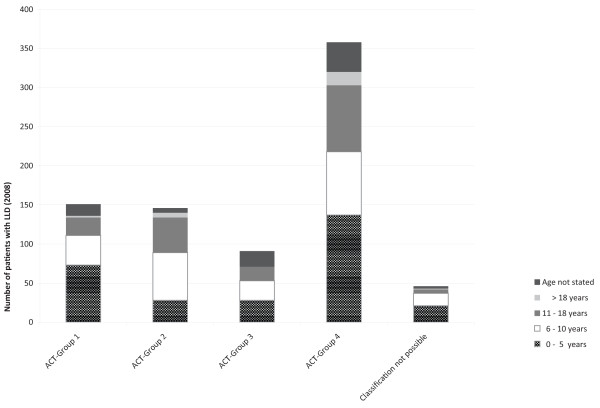
**Children cared for by paediatricians in 2008.** Illustration of reported cases of life-limiting diseases (LLD) by ACT group and age (Total: 792).

Patients were in the following age groups: 0 to 5 years, 287 (40.4%); between 6 and 10 years, 221 (31.1%); 11 to 18 years, 176 (24.8%); and older than 18 years, 26 (3.7%) (no data: 82, 8.4%). Figure 1 illustrates the reported cases of life-limiting conditions by ACT group and age.

### Disposition to commit oneself to palliative medicine

Asked if they would be disposed to engage (further) in palliative home care for children and adolescents, more than half (74, 52.5%) of the paediatricians said they would, while 66 (46.8%) did not want to be involved.

#### Interdisciplinary and trans-sectional professional networking

Nearly all participants affirmed the existence of local specialist paediatric palliative care providers; 89 (63.1%) of them reported 1 to 3 such providers, and 37 (26.2%) named 4 or more. Mostly a local children’s hospital was mentioned, followed by a paediatric home care nursing service, a psychotherapist, and a children’s hospice. Limited knowledge was reported about paediatric home hospice services and parents’ associations (Table 3).

**Table 3:** Reasons for joint patient care and participating disciplines (2008)

**Local specialist paediatric palliative care providers**	**Paediatricians aware of this service**	**Paediatricians stating joint patient care with this service**	**Reasons given for initiating a collaboration with other paediatric palliative care providers**		
			**Supportive therapy (e.g., intravenous medication)**	**Symptom control/pain therapy**	**Psychosocial support**
Children’s hospital	129 (91.5%)	108 (76.6%)	72 (51.5%)	58 (41.1%)	44 (29.1%)
Paediatric home care nursing service	110 (78.0%)	94 (66.7%)	41 (29.1%)	38 (27.0%)	51 (36.2%)
Psychotherapist	71 (50.4%)	42 (29.8%)	0	1 (0.7%)	34 (24.1%)
Children’s hospice	62 (44.0%)	37 (26.6%)	10 (7.1%)	17 (12.1%)	28 (19.9%)
Paediatric home hospice service	29 (20.6%)	11 (7.8%)	0	0	9 (6.4%)
Parents' association	31 (22.0%)	8 (5.7%)	1 (0.7%)	0	8 (5.7%)
Total	138 (97.9%)	126 (89.4%)	84 (59.6%)	78 (55.3%)	101 (71.6%)

Almost 90% of the participants collaborated with local specialist paediatric palliative care providers for the provision of care, more than three-quarters of them with a children’s hospital, and two-thirds with a paediatric home care nursing service. The pattern of networking for joint patient care between general paediatricians and local specialist paediatric palliative care providers is listed in Table 3.

As reasons for initiating collaboration with other care providers, most paediatricians (101, 71.6%) mentioned psychosocial support for the patient and his family, which was most frequently provided by a paediatric home care nursing service. Further reasons for collaborating with other local paediatric palliative care providers were supportive therapy such as intravenous medication and other invasive procedures, and the necessity of symptom and/or pain control (Table 3).

Fifty-two of the paediatricians (36.9%) attested to problems during the transition from inpatient to outpatient paediatric palliative care. The main problem was poor information flow between the children’s hospital, parents and general paediatricians (42, 29.8%), especially regarding the preparation of patients and paediatricians for crisis intervention (15.6%). Other documented factors included a lack of specialists (17, 12.1%).

#### Barriers to and incentives for the implementation of palliative home care

Palliative home care being a time-consuming occupation was perceived as a relevant challenge by 41 (29.6%) of the participants. Thirty-six (25.5%) of the general paediatricians complained about the lack of opportunities to exchange information with colleagues. Other perceived considerable hurdles are: discontinuity of care due to a temporary referral to a specialised service, such as specialist outpatient clinics (22, 15.6%); feeling overwhelmed by being solely responsible for a palliative situation (19, 13.5%); lack of adequate reimbursement (18, 12.8%); and the additional administrative burden 17 (12.1%).

Most of the participants (123, 87.1%) considered the availability of local specialist services as a favourable facilitation for the implementation of paediatric palliative home care. Education in basic palliative medicine was favoured by 119 (84.4%) and a sufficient information exchange with other care providers by 116 (82.3%) of the general paediatricians. A 24/7 on-call telephone service for paediatric palliative care was rated as an important incentive by 105 (74.5%) participants.

Issues that would be addressed most frequently to the on-call service included pain control (22, 15.6%) and medication (13, 9.2%).

## Discussion

In this study, the current status of palliative home care provided by general paediatricians in a territorial area has been analysed. The results provide relevant information on the paediatricians’ needs and concerns. The findings also highlight areas of care provision that need further improvement. Therefore, the data contribute to the evidence on needs in paediatric palliative care, in particular for areas with low patient numbers and long distances.

### Experience with paediatric palliative care

Most general paediatricians have had experience with palliative care situations in their professional practice. These findings are different from the results evaluated in NRW, which reported that less than half of the participants had such experiences [10]. This can be explained by the fact that the original instrument used in NRW was upgraded in this study: in addition to providing the WHO-definition of palliative care [25], in the questionnaire used in this study the ACT classification was introduced. This helped the participating paediatricians to correctly identify and classify their palliative care experience and is also the reason for a tenfold higher average number of patients per participant in Lower Saxony.

When extrapolating the number of patients reported by the paediatricians in this study to the population of Lower Saxony, a prevalence of about 2500 children can be assumed. This is in line with the data reported in international studies, assuming a prevalence of life-limiting conditions of 0.1% to 0.15% in children and adolescents [13,18,26]. Due to the lack of information systems to accurately identify and register children with life-limiting conditions, the latter is based on the incidence of death rather than diagnosis, which might have an adverse effect on comparability. Although the generated data can only serve as an approximate value, this study generated the first data on the prevalence of life-limiting conditions in Lower Saxony, including a classification of age and disease groups. The information on the eligible population is essential for an effective advancement of paediatric palliative care [27].

The majority of children reported by the paediatricians in Lower Saxony suffered from a condition classified as ACT group 4 (45.2%), mainly severe cerebral palsy. In contrast, the bigger proportion of children reported by the participants in NRW suffered from a condition of ACT group 1 (43.8%), nearly all of them related to malignant diseases [10]. These significant differences indicate that it seems easier for paediatricians to recognise the need for palliative care when children are in the terminal phase of malignant diseases rather than suffering from cerebral palsy, chromosomal disorders, cystic fibrosis, or muscular dystrophy [28]. Our results suggest that the validity and reliability of data can be enhanced by choosing a question format that provides information about the possible range of life-limiting conditions, including non-malignant diseases.

These observations reflect the general debate on palliative care for children suffering from illnesses other than malignancies, which remains controversial [29]. Our results are in line with recent studies on palliative care for adults, finding that most generalists defined palliative care narrowly in terms of the very end of life and typically within the context of cancer [5], and that palliative care needs of non-cancer patients are often neglected [4]. But life-limiting conditions as per ACT groups 2 to 4 define the majority of paediatric patients in a palliative situation [30]; they are in many cases diagnosed at birth or early in life, and the courses of these diseases are highly unpredictable, most often emerging within the context of a diverse set of complex chronic conditions [31].

For all (potentially) life-limiting conditions the relationship between cure and palliation should not be considered as mutually exclusive, because a neglect of treatment options with palliative intentions can cause unmet needs of patients and families [32]. The more secure the attitude of general paediatricians towards this morally and medically challenging topic, the better the quality of the individually provided health care will be. The lack of understanding of palliative care amongst generalists is seen as a barrier to good palliative care provision for adults [5]. Our findings suggest that the general paediatricians’ expertise on the various aspects of paediatric palliative care needs expansion to assure appropriate support for paediatric patients and families, especially when dealing with diseases other than cancer. The perception of paediatric palliative care as a philosophy that is integral to the paediatrician’s practice could be achieved by the assistance of paediatric palliative care specialists and with professional exchange. Therefore, the best practice in specialist support is described as ranging from on-off consultation to full referral with the primary care team usually determining the need of support [33]. In areas with a predominantly rural infrastructure the assistance of palliative care specialists can best be realised by developing a systematic mechanism for sharing resources and providing consultation [34].

### Interdisciplinary and trans-sectoral professional networking

Psychosocial support being the most striking reason for initiating collaboration with other care providers was also evaluated for general practitioners in palliative care for adults [35].

Within the paediatricians’ awareness of the existence of other paediatric palliative service providers, paediatric hospice home services and parents’ associations were clearly underrepresented when compared to children's hospitals or home care nursing services.

Communication and cooperation with medical staff seemed more obvious to paediatricians than with hospice services or parents’ groups.

The detected lack of recognition of hospice services could also be a result of the paediatricians’ unwillingness to accept the incurable condition of the child [21] and also of the fear of the word “hospice” as a generally understood synonym for “taking away hope” [36].

Temel et al. recently provided evidence that palliative care significantly improves the quality of life, mood, and life expectancy of adult cancer patients [37]. Even if this has to be verified for paediatric patients, it might be assumed that paediatric palliative care will enhance the situation of children and adolescents suffering from life-limiting conditions. This potential benefit of paediatric palliative care has to be accessible for all children suffering from life-limiting conditions.

The mission of palliative care teams to meet the physical, psychological, emotional, spiritual, and social needs can best be accomplished by multi-professional cooperation [1]. The lack of familiarity with the availability and suitability of hospice services and parents’ groups is an important barrier for their involvement when potentially appropriate [22]. To assure a good communication flow, central coordination achieved by the appointment of a primary professional in charge is recommended [38]. Based on these previous findings, the results of our study underline that adequate collaboration and specialised education of care providers are necessary to optimise the multi-professional cooperation in paediatric palliative care.

Concerning the transition from inpatient to outpatient paediatric palliative care, the information flow and communication, especially between children’s hospital staff and both general paediatricians in private practices and parents, need improvement, because high-quality paediatric palliative care also depends on trans-sectoral linkages [23,39]. Hospital-based palliative care consultation services seem to be very appropriate in this regard, since they can provide support to the paediatrician while strengthening the relationships between the hospital staff and community service providers [11].

### Barriers to and incentives for the implementation of palliative home care

In line with previous studies [7,16,21], time demand and lack of exchange in a team are the most prominent difficulties providing palliative home care in Lower Saxony.

The known lack of clearly defined legal and financial regulations is a significant obstacle for general paediatricians in the home care setting [7]; therefore, a clear legal regulation of general palliative home care is needed. This demand was already expressed in other countries [38]. Since April 2007, German social legislation has provided the individual the right to specialist palliative home care for all people with statutory health insurance. General palliative home care, as provided by general paediatricians, is not regulated yet and certainly needs advancement [40].

Besides the availability of a specialist paediatric care consultant team, education in basic palliative competence in this study was requested most for the implementation of paediatric palliative home care. In this regard, a small expenditure of time can already produce appreciable results in reassuring paediatric residents [41] and can be tailored to particular regional needs [42].

A noteworthy number of participants used the free-text option to express a need for support in pain therapy. The physicians’ uncertainty in this regard has already been described before [15]. Many children experiencing life-limiting conditions suffer from pain at the end of their life [43], and pain relief is often insufficient [13,44,45]. Optimal pain control might therefore be mainly provided by specialists such as anaesthesiologists with special training in pain control, since general paediatricians often lack sufficient experience in this field.

The provision of palliative home care for adults is a special challenge within a predominantly rural infrastructure [46,47]. Because of the lower case numbers, it is even more difficult to offer a comprehensive specialist palliative home care for children and adolescents in areas with low population density and long distances; this can be met with the integration of existing structures, the bundling of competences, and good networking. Therefore, the cooperation of specialists and general paediatricians is crucial.

As for the general limitations of this study, collecting data for organisational reasons took place over a time period of ten months. Within this period, palliative care in Germany developed considerably, which might have partly affected the interpretation of our findings.

The paediatricians completed the questionnaire on an evening session of a quality circle, which probably had a negative impact on completeness and correctness, especially respective to the details on their patients.

## Conclusions

Our findings suggest that cooperation between palliative care providers and paediatricians could be improved. Good information flow and coordination are key issues in this regard.

## Availability of supporting data

The used questionnaire is available as Additional file 1 with the manuscript. For further questions, please contact the authors.

## Endnote

Analysis of variance (ANOVA), level of significance: 0,05.

## Competing interests

The authors declare that they have no competing interests.

## Authors’ contribution

KK participated in the conception, design, analysis, interpretation, and drafting of the manuscript. NE participated in the conception, design, analysis, interpretation, and revision of the manuscript. SJ participated in the conception, design, analysis, interpretation, and revision of the manuscript. AS participated in the conception, design, analysis, interpretation, and revision of the manuscript. MG participated in the conception, design, analysis, interpretation, and revision of the manuscript. DR participated in the conception, design, analysis, interpretation, and revision of the manuscript. All authors read and approved the final manuscript.

## Supplementary Material

Additional file 1 Questionnaire on Paediatric Palliative Home Care by General Paediatricians in their own practice. Click here for file
