# Supplementary material for: Paediatric palliative home care in areas of Germany with low population density and long distances: a questionnaire survey with general paediatricians
Source: BMC Res Notes. 2012 Sep 11;5:498. doi: 10.1186/1756-0500-5-498 (PMC3532334; doi:10.1186/1756-0500-5-498)
Supplement: Additional file 1 — Questionnaire on Paediatric Palliative Home Care by General Paediatricians in their own practice. [file 1756-0500-5-498-S1.doc]

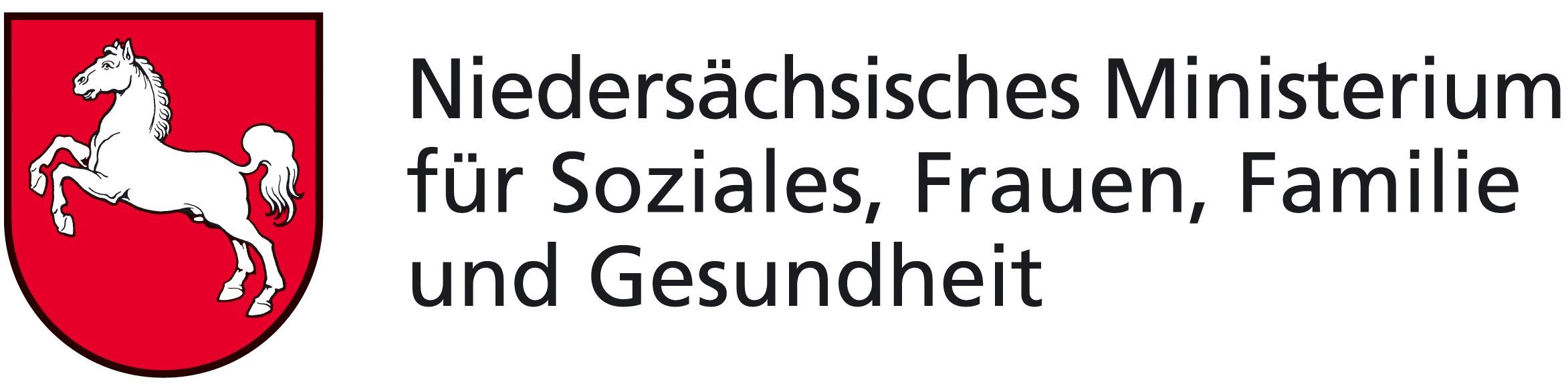


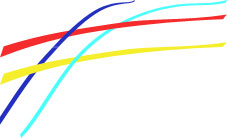


Netzwerk für die Versorgung schwerkranker Kinder und Jugendlicher e.V.

Funded by:

**Paediatric Palliative Home Care**

**by General Paediatricians in their own practice**

**in Lower Saxony**

**Questionnaire**

**
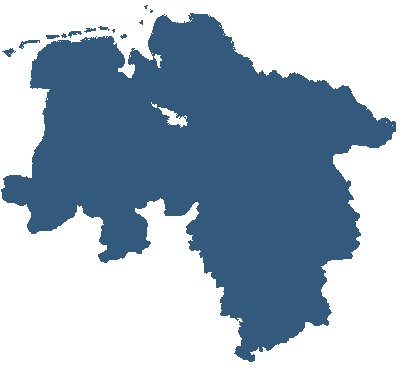
**

**
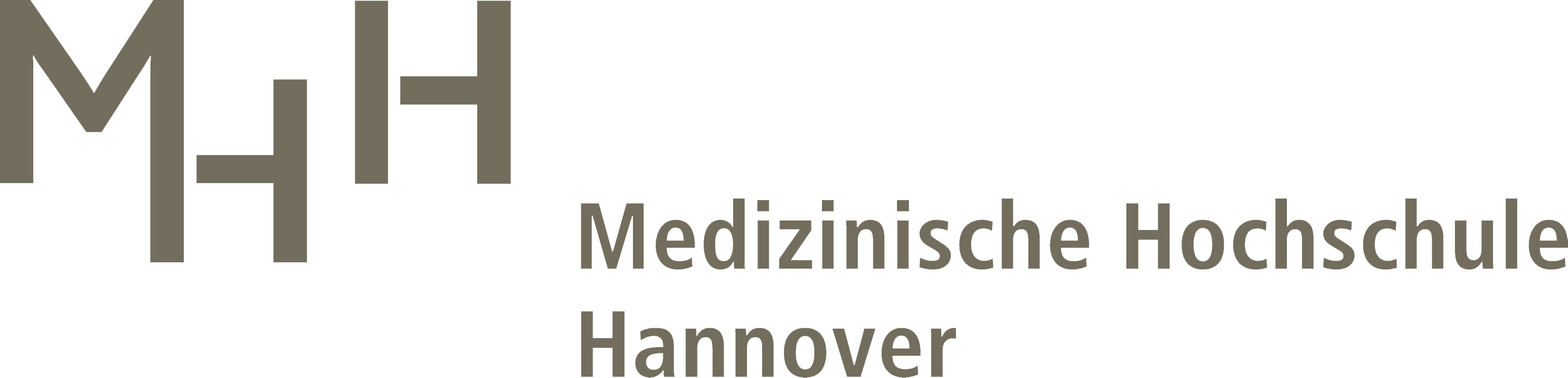

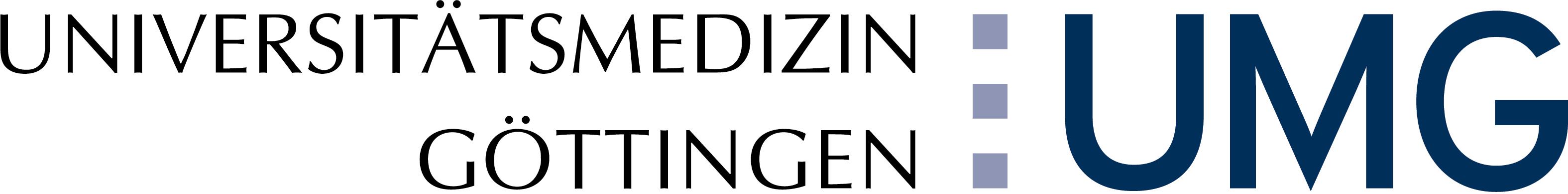
**

**Questionnaire on Paediatric Palliative Home Care by General Paediatricians in their own practice**

The federal state of Lower Saxony pursues the objective to advance home care for children and adolescents with a severe life-limiting disease. In order to achieve a targeted and sensible improvement of care provision, information has to be gathered about the present care situation. *Your* experience and *your* point of view are the prerequisite for a further development of care delivery. We ensure that the questionnaires will be dealt with confidently; data processing and analysis will occur anonymously.

In the following questions, *paediatric palliative care* will be used in accordance with the WHO definition and refers to the care for children and adolescents suffering from a life-limiting disease with the aim of improving their quality of life. It begins when illness is diagnosed and continues regardless of whether or not a child receives treatment directed at the disease. Palliative care can mean the accompaniment of a patient until the end of his life but is not restricted to this phase.

## Questions on previous experience with life-limiting conditions

**1. Do you care for children with life-threatening or life-limiting diseases in your own practice?**

yes **If yes, please proceed to question 2**.

no **If no, please proceed to question 6.**

**2. How many children did you take care of in the year 2008 because of a life-threatening or life-limiting disease?**

0 – 5  6 – 10  11 – 20  ≥ 21

**3. To which group did these diseases belong?** (multiple answers possible)

1. Conditions for which treatment with curative intention is feasible but might fail;

e.g., cancer, irreversible organ failure.

2. Premature death is anticipated, prolonging good-quality life is possible;

e.g., cystic fibrosis, muscular dystrophy.

3. Progressive conditions, no curative therapy possible;

e.g., metabolic disorders.

4. Irreversible, non-progressive diseases, complications presumed;

e.g., severe cerebral palsy, chromosomal disorders.

**4. Please note diagnoses, the number, and the age of children with life-threatening or life-limiting diseases you took care of in 2008.**

**Condition Number Age**

e.g., cystic fibrosis |_3| thereof: |__| 0 – 5 |_2| 6 – 10 |_1| 11 – 18 |__| > 18

**_________________** |__| thereof: |__| 0 – 5 |__| 6 – 10 |__| 11 – 18 |__| > 18

**_________________** |__| thereof: |__| 0 – 5 |__| 6 – 10 |__| 11 – 18 |__| > 18

**_________________** |__| thereof: |__| 0 – 5 |__| 6 – 10 |__| 11 – 18 |__| > 18

**_________________** |__| thereof: |__| 0 – 5 |__| 6 – 10 |__| 11 – 18 |__| > 18

**5. Possible additions on question 4.**

**e.g., type or duration of care**

**___________________________________________________________________________**

**Questions on exposure to situations requiring paediatric palliative care, professional networking, and the transition from inpatient to outpatient care**

**6. Have you already taken care of dying children?**

yes **If yes, please proceed to question 7.**

no, never **If no, please proceed to question 8.**

**7. If yes, how many dying children have you taken care of?**

In 2008:|__|__|

In 2006 and 2007: |__|__|

In 2005 or earlier: |__|__|

**8. Would you generally be disposed to engage (further) in palliative home care for children and adolescents?**

yes, definitively  rather no

rather yes no, definitely not

**9. What local specialised paediatric palliative care providers do exist in your catchment area?**

(multiple answers possible)

Children’s hospital  Paediatric home care nursing service  Paediatric home care hospice service

Children’s hospice  Paediatric psychotherapist  Parent’s association

Other. Please specify: _____________________________________________________

___________________________________________________________________________

**10. Which specialised paediatric palliative care providers did you cooperate with in 2008?**

(multiple answers possible)

**Information exchange Joint**

**e.g., round table patient**

**care**

- Children’s hospital
- Paediatric home care nursing service
- Paediatric psychotherapist
- Children’s hospice
- Paediatric home care hospice service
- Parents’ association
- _______________

**11. What were the reasons for cooperating?** (multiple answers possible)

**Supportive therapy Palliative Psychosocial**

**e.g., intravenous**  **symptom control/ support**

**medication pain therapy patient/family**

- Children’s hospital
- Paediatric home care nursing

service

- Paediatric psychotherapist
- Children’s hospice
- Paediatric home care hospice

service

- Parents’ association
- _______________

**12. Were there other reasons for cooperating? Please specify:**

___________________________________________________________________________

___________________________________________________________________________

___________________________________________________________________________

___________________________________________________________________________

**13. Are there problems during the transition from inpatient to outpatient paediatric palliative care?**

yes **If yes, please proceed to question 14.**

no **If yes, please proceed to question 15.**

**14. Which problems exist during transition?** (multiple answers possible)

Poor information flow

Lack of local specialists (e.g., paediatric home care nursing service)

Incomplete formulation by children’s hospital

Poor information flow between children’s hospital and parents for the case of crisis intervention

Poor information flow between children’s hospital and paediatricians for the case of crisis intervention

Other. Please specify_____________________________________________________

**Barriers to the implementation of paediatric palliative home care**

**15. The following items refer to potential barriers that you perceive or have already experienced with respect to the implementation of paediatric palliative home care. Please evaluate the extent to which each of these barriers – according to you – impedes the implementation of care delivery.**

**No problem Insuperable**

**at all problem**

- Formalities (forms, prescriptions, applications)
- Financial burden (budget)
- Time demand (e.g., home visits)

**No problem Insuperable**

**at all problem**

- Emotional burden caring for dying children
- Professional uncertainty with respect to prognosis
- Lack of special knowledge with respect to appropriate medical care

(diagnostics, interventions, medication, etc.)

- Uncertainty towards patient and family
- Lacking continuity of contact due to temporary treatment of the child/adolescent in specialist outpatient clinics/inpatient units.
- Sole responsibility
- Lack of exchange with a team
- Other. Please specify: ____________________

**____________________**

**Incentives to the implementation of paediatric palliative home care**

**16. The following items refer to types of support that you appreciate as helpful with respect to palliative home care for children and adolescents. Please evaluate the extent to which each of these options – according to you – facilitates the implementation of care delivery.**

**Not Very**

**helpful at all helpful**

- Training/education in communication skills
- Training/education in basic palliative care competence

- Adequate remuneration
- Availability of specialist supportive services in local proximity (e.g., paediatric nursing home care service, paediatric volunteer hospice service)
- Opportunity for professional exchange

(e.g., in a case conference)

- 24h on-call service for accessibility of a specialist

or consulting team

- Regular information if the patient is under medical

treatment of specialists in the meantime.

- Other. Please specify: ____________________

____________________

1. **If a 24h on-call service is desired, for which issue would you need support for optimal paediatric palliative care?**

____________________________________________________________________________

____________________________________________________________________________

____________________________________________________________________________

_________________________________________________________________________ ___

**Demographic data**

**18. Personal details:**  Woman Age in years |__|__|

Man

**19.** **For how long have you been settled in your own practice?**  Month |__|__| Year |__|__|__|__|

**20. What kind of practice do you run?**

own practice  joint practice

**21. Where is your practice located?**

(rather) rural  (rather) urban

**22. How many patients do you treat per quarter?** |__|__|__|__|

**23. Have you completed a course in palliative medicine?**  yes  no

**Are you at present completing a course in palliative medicine?**  yes  no

**Do you plan to complete a course in palliative medicine?**  yes  no

**24. Do you hold available prescriptions for anaesthetics?**

yes  no

**If yes, how often did you prescribe anaesthetics in 2008?** __________

**Suggestions and Supplementary Notes**

**25. Do you have supplementary notes or suggestions concerning the questionnaire?**

**Are there important aspects of paediatric palliative care that were not/inadequately taken into account?**

**We appreciate every suggestion**.

_____________________________________________________________________________

_____________________________________________________________________________

_____________________________________________________________________________

_____________________________________________________________________________

_____________________________________________________________________________

_____________________________________________________________________________

_____________________________________________________________________________

_____________________________________________________________________________

**Thank you very much for your support!**

Netzwerk survey 2009 Date |__|__||__|__||__|__| ID-Number: |__|__|__|

The ID-Number is used to control the response rate. Anonymity is kept in any case.
